# X-ray phase microtomography with a single grating for high-throughput investigations of biological tissue

**DOI:** 10.1364/BOE.8.001257

**Published:** 2017-01-31

**Authors:** Marie-Christine Zdora, Joan Vila-Comamala, Georg Schulz, Anna Khimchenko, Alexander Hipp, Andrew C. Cook, Daniel Dilg, Christian David, Christian Grünzweig, Christoph Rau, Pierre Thibault, Irene Zanette

**Affiliations:** 1 Diamond Light Source, Harwell Science and Innovation Campus, Didcot, Oxfordshire OX11 0DE, UK; 2 Department of Physics & Astronomy, University College London, London WC1E 6BT, UK; 3 Institute for Biomedical Engineering, ETH Zürich, 8092 Zürich, Switzerland; 4 Biomaterials Science Center, Department of Biomedical Engineering, University of Basel, 4123 Allschwil, Switzerland; 5 Helmholtz-Zentrum Geesthacht, 21502 Geesthacht, Germany; 6 University College London Institute of Cardiovascular Science, London WC1E 6BT, UK; 7 Paul Scherrer Institut, 5232 Villigen PSI, Switzerland; 8 School of Materials, University of Manchester, Manchester M1 7HS, UK; 9 Department of Otolaryngology, Northwestern University, Feinberg School of Medicine, Chicago, Illinois 60611, USA; 10 Department of Physics & Astronomy, University of Southampton, Southampton SO17 1BJ, UK

**Keywords:** (170.3880) Medical and biological imaging, (170.6960) Tomography, (340.7450) X-ray interferometry, (110.7440) X-ray imaging, (180.7460) X-ray microscopy

## Abstract

The high-throughput 3D visualisation of biological specimens is essential for studying diseases and developmental disorders. It requires imaging methods that deliver high-contrast, high-resolution volumetric information at short sample preparation and acquisition times. Here we show that X-ray phase-contrast tomography using a single grating can provide a powerful alternative to commonly employed techniques, such as high-resolution episcopic microscopy (HREM). We present the phase tomography of a mouse embryo in paraffin obtained with an X-ray single-grating interferometer at I13-2 Beamline at Diamond Light Source and discuss the results in comparison with HREM measurements. The excellent contrast and quantitative density information achieved non-destructively and without staining using a simple, robust setup make X-ray single-grating interferometry an optimum candidate for high-throughput imaging of biological specimens as an alternative for existing methods like HREM.

## 1. Introduction

The three-dimensional visualisation of biological tissues is the basis for a large number of biomedical studies, such as investigations of diseases and developmental defects. For this, it is required to obtain high-contrast, high-resolution images of the specimens in a reasonably short time and without the need for elaborate sample preparation.

Several techniques have been developed for the investigation of biological samples. Histology [1,2] – for long the gold standard for tissue investigation – can achieve excellent 2D resolution and contrast, but is a destructive technique, needs extensive sample preparation including staining agents and suffers from tissue deformations making registration of slices and hence 3D reconstruction of the volume cumbersome. Drawbacks of other available methods include the need for staining procedures (absorption X-ray micro computed tomography in combination with contrast agents [3, 4]), the limitation in spatial resolution (micro magnetic resonance imaging [5,6]), the requirement of transparency and small size of the sample (optical projection tomography [7,8]) and a relatively low contrast and limited penetration depth (optical coherence tomography [9]). High-resolution episcopic microscopy (HREM) has recently seen increased interest, in particular for imaging of embryos in developmental studies [10–13]. The technique is based on automated physical sectioning of the sample embedded in a resin in layers of a few micrometers thickness [10]. After each slicing step, the surface of the sample block is imaged with a visible light microscope and a volume data set might then be obtained by combining together the slice series.

Most of the drawbacks of the commonly employed methods can be overcome by X-ray phase-contrast imaging (XPCI), which delivers high-contrast, high-resolution three-dimensional information about the density composition of the sample in a non-destructive way and without the need for contrast agents [14,15]. The technique uses the phase shift of X-rays induced by the sample to generate image contrast. The interaction of X-rays with the sample can be described by the complex refractive index *n* = 1 − *δ* + *iβ*, in which *δ* and *β* account for the phase shift and absorption in the specimen, respectively [16]. For biological soft tissues with small density differences, the refractive index decrement *δ* can be up to three orders of magnitude larger than the imaginary part *β* of the refractive index and XPCI can deliver images with much higher contrast compared to conventional absorption imaging in these cases.

Among the different XPCI methods, X-ray grating interferometry (XGI) [17–19] achieves excellent contrast and quantitative information about the density distribution of biological soft-tissue samples when employed in tomographic mode [20–23]. The potential of XGI for histopathological, diagnostic, or structural and morphological studies of soft tissues with high contrast and high resolution has been evaluated against a range of other commonly used methods such as conventional absorption-based microtomography [24] and mammography [25], magnetic resonance imaging [26], histology [27] and cryotome-based planar epi-illumination imaging [28], while a direct comparison with the state-of-the-art HREM technique has not been reported until now.

The conventional implementations of XGI at synchrotrons use a setup with a beam-splitter phase grating and an absorption grating that converts the high-frequency interference pattern into intensity modulations in the detector pixels while one of the gratings is stepped perpendicular to the grating lines, known as phase-stepping scan. A simpler and more robust setup can be achieved with the use of only a single phase grating, which has been reported for single-shot measurements with 2D and 1D gratings [29–31]. Recently, a setup using the phase-stepping approach with a single line grating has been proposed allowing more dose- and time-efficient imaging with a simple arrangement albeit a decreased spatial resolution [32].

Here, we present the first implementation of XGI with a single grating for biological imaging at the I13-2 Diamond-Manchester Branchline at Diamond Light Source (DLS), UK and we compare the reconstructed phase volumes with results obtained by the state-of-the art HREM technique, demonstrating the high potential of single-grating XGI for quantitative biological imaging.

## 2. Materials and methods

### 2.1. X-ray single-grating interferometry

At synchrotrons, X-ray grating interferometry is commonly implemented using two gratings: a beam-splitter phase grating, which creates a high-contrast interference pattern with a period in the *μ*m-range used as reference, and an absorption grating, which is needed to analyse this pattern when it cannot be resolved by the detector [19]. However, the absorption grating is no longer strictly required if the interference pattern can be directly resolved, which is achieved by using a beam-splitter phase grating with a sufficiently large period and a high spatial resolution detector system [32]. The single-grating setup is simpler and more robust than the conventional use of two gratings as it does not require alignment. Furthermore, it is more dose-efficient and can provide a better signal-to-noise ratio, as the analyser grating, which absorbs a significant part of the X-rays in common two-grating interferometry, is absent.

Generally, a phase grating with larger period will be chosen for the single-grating implementation as the period of the interference pattern needs to be resolved by the detector. This affects both the spatial resolution and the angular sensitivity of the measurement. The spatial resolution of the images is limited by the detector resolution and ultimately by the separation 2*λz/p* of the first order diffracted beams created by the phase grating, where *λ* is the wavelength, *z* the grating-detector distance and *p* the period of the phase grating [19]. Blurring of the image occurs when the detector resolution is better than the beam separation. The angular sensitivity is inversely proportional to the period *p* of the grating and scales with the propagation distance *z* [33]. In practice, *z* as well as *p* have to be chosen carefully to avoid blurring from large beam separation while maintaining a high sensitivity and spatial resolution.

### 2.2. Experimental setup at Diamond I13

Measurements were conducted at the I13-2 Diamond-Manchester Imaging Beamline of the DLS [34]. The X-ray radiation is produced by a 2 m-long undulator insertion device (23 mm period) located approximately 220 m upstream of the experimental hutch. A monochromatic X-ray beam of energy 19 keV was extracted with a silicon (111) double crystal monochromator for the measurements.

**Fig. 1:**
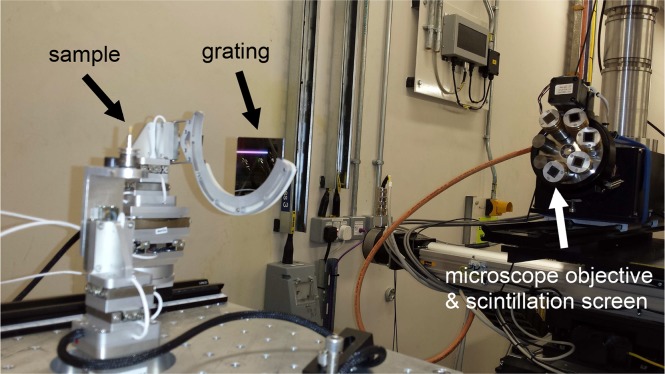
Experimental setup using a single phase grating (period: 10 *μ*m) mounted at a distance of 5 cm downstream of the sample. A high-resolution X-ray detector (effective pixel size: 1.13 *μ*m) is placed 72 cm further downstream to record the interference pattern created by the grating. The phase shift of the X-ray wavefront induced by the sample results in a distortion of the interference pattern.

The results presented here were obtained with the single-grating XGI setup shown in Fig. 1. The sample was mounted on a high-precision rotation stage and the phase grating was placed approximately 5 cm downstream. The grating was fabricated at the Institute for Microstructure Technology (IMT) of the Karlsruhe Institute of Technology (KIT) using LIGA-processing [35]. It was made of nickel and had a nominal period of *p* = 10 *μ*m. The grating lines were 10.4 *μ*m high, which yields a phase shift of 3*π*/2 at an energy of approximately 20 keV. The detector system was positioned at a distance of *z* = 72 cm downstream, where the interference pattern produced by the grating showed maximum contrast and had the same period as the grating itself. The detector system was assembled in-house and consisted of a scintillation screen coupled to an infinity-corrected optical microscope and a pco.4000 CCD camera (chip size: 4008 × 2672 pixels, pixel size: 9 *μ*m). The scintillation screen was a double-side polished CdWO_4_ crystal of 150 *μ*m thickness supplied by Hilger Crystals Ltd. A 4× objective (Olympus UPLSAPO 4X) with a numerical aperture of 0.16 was used in combination with a tube lens and a relay optics system to achieve optimal imaging performance over the large chip of the pco.4000 CCD camera. This led to an 8-fold total optical magnification and an effective pixel size of 1.13 *μ*m. The resolution of the detector system at 10% MTF (modulation transfer function), determined from a star test pattern measurement, is approximately 433 cycles/mm, equivalent to a smallest resolvable feature size of about 2.3 *μ*m.

### 2.3. Sample preparation and choice

The potential of XGI with a single grating for biological imaging was evaluated on a wild-type mouse embryo (strain C57BL/6N) at estimated embryonic day (ED) 13.5 with the head removed.

Animal maintenance, husbandry and procedures were carried out in accordance with British Home Office regulations (Animals Scientific Procedures Act 1986).

The embryo was fixed overnight in 4% paraformaldehyde (PFA) in phosphate buffered saline (PBS) and then dehydrated by increasing concentration of ethanol (30% to 100%). It was then cleared using histoclear (National Diagnostics) for one hour, and embedded in paraffin overnight after several wax washes at 60 °C. The paraffin was trimmed down to a small cylinder around the sample. For the measurements the paraffin cylinder was mounted on a translation and tomographic rotation stage as shown in Fig. 1.

Mice are often used as models to study embryonic development and disorders because they are genetically and physiologically similar to humans, their genes can be easily manipulated and they reproduce fast [36,37]. Recently, mice have been used extensively as model animal systems for genetic knock-out studies, where a certain gene of interest is turned off, in order to learn more about its function by comparison with wild-type individuals. One aim of these studies is to identify lethal gene knock-outs to understand the significance and function of genes critical for embryonic survival and normal development [38–40]. The information from the mouse model investigations can then help to give insight into the causes of human congenital abnormalities to develop methods for early diagnosis and therapy [40,41].

For these types of studies, high-throughput imaging of a large number of specimens is essential and it is important to visualise the inner structure of the embryos with high contrast and high resolution to detect abnormalities. In the following, we demonstrate that XGI with a single grating is a suitable candidate for this purpose and shows advantages over existing methods.

### 2.4. Data acquisition and analysis

Data acquisition and analysis for the single-grating setup can be carried out following the same Fourier analysis approach as in conventional two-grating interferometry [19]. Commonly, a phase-stepping scan is performed by laterally scanning the grating in the direction transverse to the grating lines in small steps over at least one period of the interference pattern. The phase-stepping curves are sequentially acquired with (sample scan) and without (reference scan) the sample in the beam. The recorded intensity oscillation in each pixel can be approximated as a sinusoidal curve, e.g. in frequency space by considering only the zeroth and first order components of the Fourier series. From these raw data, three different image signals can be reconstructed, namely transmission, differential phase and dark-field [42].

The transmission signal, similar to the signal measured without the grating in the beam, is given by the ratio of zeroth order Fourier coefficients of the sample and reference scan. The differential phase shift *∂*Φ/*∂x* perpendicular to the grating lines is directly related to the refraction angle *α* in the horizontal direction, which is given by the phases *ϕ_s_* and *ϕ_r_* of the sample and reference intensity curves, the grating period *p*, the grating-detector distance *z* and the X-ray wavelength *λ*:

(1) $$\frac{\partial \Phi }{\partial x}=\frac{2\pi }{\lambda }\alpha =\frac{p}{\lambda z}(\phi_{s}-\phi_{r}),$$

Furthermore, the dark-field signal can be obtained from the same data set, which quantifies the amplitude reduction of the intensity oscillations due to small-angle scattering when the specimen is in the beam [42]. As this signal is not of relevance for the sample presented here, it is not further discussed.

Phase stepping of the grating was performed in 5 steps over one grating period, and the acquisition time was 2 s per frame. A total of 1201 sample scans were acquired for evenly spaced viewing angles of the sample over 180 degrees rotation and reference scans were taken every 200 angles. The total exposure time of the tomography scan was approximately 3.5 hours including overhead time due to motor movement and data transfer.

From each phase-stepping scan, a differential phase and a transmission signal were retrieved by Fourier analysis. The phase volume was reconstructed from the differential phase projections using the filtered back-projection (FBP) algorithm with a Hilbert filter, which incorporates the integration step [43]. The phase shift Φ is related to the refractive index decrement *δ* via:

(2) $$\Phi =\frac{2\pi }{\lambda }\int \delta dz,$$

where the integral denotes integration over the sample thickness in beam direction *z*. Hence, multiplication of the phase volume with *λ*/2*π* gives directly the 3D distribution of *δ* in the sample. For the investigation of biomedical specimens, the electron density *ρ_e_* is the typical quantity of interest. It is in good approximation directly proportional to *δ* for materials containing low-Z elements at the given photon energy [44]:

(3) $$\rho_{e}=\frac{2\pi }{r_{0}\lambda^{2}}\delta ,$$

where *r*_0_ is the classical electron radius. Therefore, a 3D electron density map of the sample can directly be derived from the phase volume. Moreover, *ρ_e_* is approximately proportional to the mass density of the object, so that the phase volume gives information about the physical density properties of the specimen.

From the same data set, also an absorption tomogram, similar to the one that would have been obtained without grating in the beam, can be retrieved from the reconstructed transmission signal. The transmission *T* can be expressed in terms of the linear attenuation coefficient *μ*, which is directly related to the imaginary part *β* of the refractive index via *μ* = 4*πβ/λ*:

(4) T=exp(−∫μdz),

with integration over *z* in beam direction. The tomographic reconstruction of the absorption volume was performed with the standard FBP algorithm including logarithmisation [45]. It should be noted that the transmission/absorption projections and volume show a signal similar to the one that would have been measured inline without the grating interferometer. Under conditions not optimised for absorption imaging, it, however, does not correspond to the pure absorption of X-rays, but is also influenced by propagation effects. In the following, we therefore refer to this signal as propagation signal.

Three-dimensional volume rendering of the mouse embryo was performed from the phase tomogram using Avizo 9.1.1 software.

## 3. Results

### 3.1. Interference pattern

**Fig. 2:**
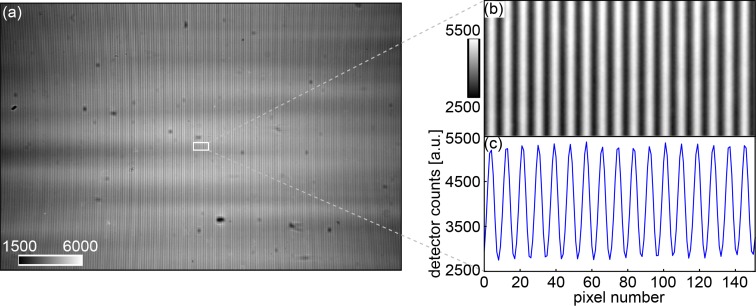
(a) Interference pattern at the first fractional Talbot distance. The visibility in the centre of the field of view is approximately 29%. (b) Region of interest (152×76 pixels) in the centre of the image. (c) Horizontal profile through (b) averaged along the vertical direction. The grey values represent arbitrary intensity units.

The interference pattern produced by the phase grating at the first fractional Talbot distance [46] is shown in Fig. 2(a). The visibility – determined via Fourier analysis as the ratio of the first and zeroth order Fourier coefficients of the phase stepping curve – is approximately 29% in the central part of the image. It is limited mainly by the lateral coherence of the X-ray beam reduced by the finite source size, roughness and instabilities of the optical components in the beam and defects of the scintillation screen.

A region of interest (ROI) of the line pattern taken from the centre of the field of view can be seen in Fig. 2(b) and a horizontal profile averaged along the vertical direction is plotted across this area in Fig. 2(c).

### 3.2. Differential phase and propagation projections

**Fig. 3:**
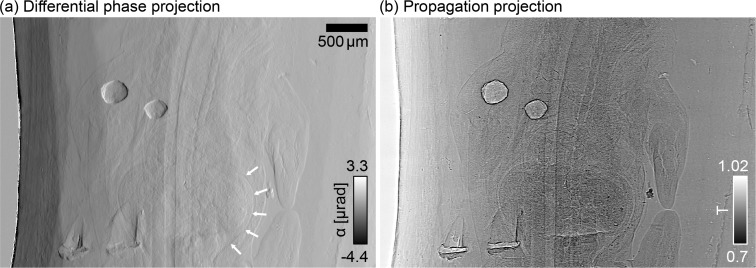
One of the 1201 reconstructed projections showing (a) the differential phase signal measured as the refraction angle of the incoming X-rays induced by the sample and (b) the propagation signal, consisting of absorption and edge enhancement effects.

From the phase stepping scans with and without sample in the beam, the refraction angle of the X-rays in the specimen and the propagation signal were reconstructed as described in the previous section for each of the 1201 viewing angles of the tomography scan. Examples of the reconstructed projections are shown in Fig. 3. In the differential phase signal in Fig. 3(a) not only the outlines of the embryo and its internal structures, such as the peritoneal cavity (indicated by white arrows), are clearly visible, but also structures within the organs can be observed. Two air bubbles in the paraffin wax lead to strong refraction. On the left side of the field of view the edge of the paraffin embedding can be seen causing some phase wrapping. The projection in Fig. 3(b) is similar to the one that would have been obtained without the grating in the beam and contains a combination of absorption and edge-enhancement effects arising upon propagation.

A typical quantity of interest to asses the quality of the phase-contrast measurement is the angular sensitivity, which is a measure of the resolution of the angular deviation measurement. It is commonly estimated from the reconstructed differential phase projections by taking the standard deviation of a region in the background area without sample. The angular sensitivity of the measurements presented here was calculated from a 150×150 pixels area in the surrounding paraffin and is approximately 110 nrad.

### 3.3. Phase volume

**Fig. 4:**
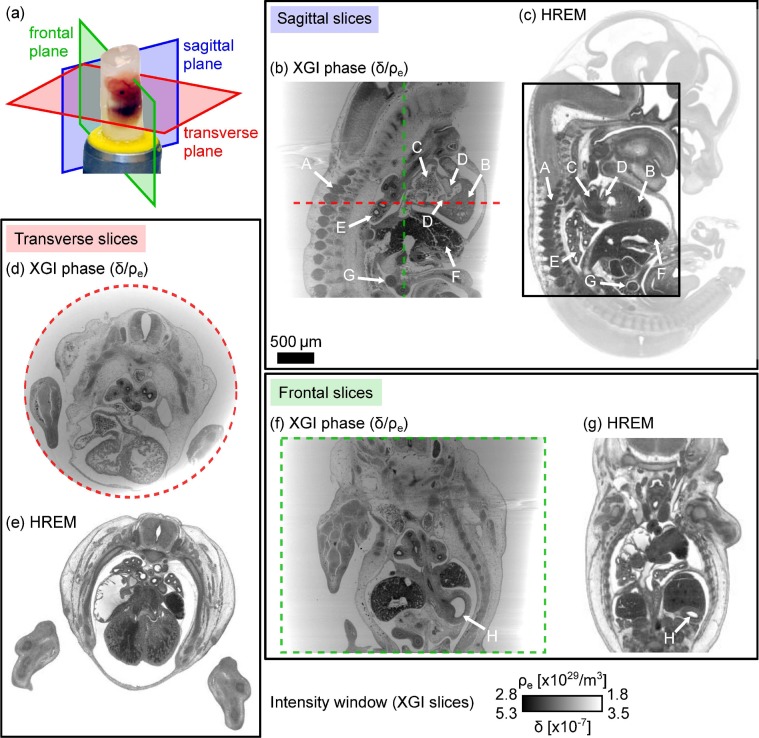
Sagittal, transverse and frontal slices through the reconstructed phase volumes of a mouse embryo (head removed) embedded in paraffin wax in panels (b), (d) and (f) compared to the results obtained with HREM of a different specimen at the same gestational stage (ED13.5) in panels (c), (e) and (g). Organs within the embryo are indicated by white arrows. Panel (a) shows the definition of the sectioning planes on a photograph of an embryo specimen. *HREM data provided by Deciphering the Mechanisms of Developmental Disorders (http://dmdd.org.uk/), a programme funded by the Wellcome Trust with support from the Francis Crick Institute, is licensed under a Creative Commons Attribution Non-Commercial Share Alike licence.*

Slices through the phase tomogram are presented in Figs. 4(b), 4(d) and 4(f). They are compared to the equivalent images of a second mouse embryo sample (with head) at the same gestational age (ED13.5) obtained with the state-of-the-art HREM method, see Figs. 4(c), 4(e) and 4(g) (*HREM data provided by Deciphering the Mechanisms of Developmental Disorders (http://dmdd.org.uk/), a programme funded by the Wellcome Trust with support from the Francis Crick Institute, is licensed under a Creative Commons Attribution Non-Commercial Share Alike licence.*).

The orientations of the presented slicing planes – sagittal, transverse and frontal – are illustrated on a photograph of a mouse embryo sample in Fig. 4(a). The phase tomogram slices shown in Figs. 4(b), 4(d) and 4(f) provide detailed insight into the inner features of the sample: not only the central part of the embryo’s body can be clearly distinguished from the surrounding paraffin using the phase information, but also the different organs are visualised with high contrast as highlighted by the labelled arrows in Figs. 4(b) and 4(f). In particular, the spine (A), the heart ventricles (B) and atria (C), the lungs (E), the liver (F), and intestines (G) of the mouse embryo can be identified and their inner structure is unveiled. Within the heart it is possible to even distinguish the atrioventricular cushions (D) from the surrounding atrial and ventricular myocardium (B, C). In the frontal slice in Fig. 4(f), the stomach (H) can be seen. The transverse slice in Fig. 4(d) reveals the fine structure of the heart ventricles. When comparing the results from XGI and HREM measurements, it can be noted that for sagittal, transverse and frontal planes all features in the HREM images can be identified in the XGI slices. Moreover, they are shown with comparable image quality in terms of contrast and resolution.

A non-uniformity of the paraffin background is visible in the phase tomogram slices in Fig. 4. This can be attributed to the effects of phase wrapping and region-of-interest tomography. Strong refraction at the air-paraffin interface causes phase wrapping at the edge of the wax block. This could be avoided, for example, by placing the sample in a water bath during the measurement. Furthermore, parts of the paraffin block moved out of the field of view for some projections of the tomography scan. This leads to a cupping artefact in the transverse slices of the reconstructed phase volume. In future studies care will be taken to avoid region-of-interest tomography by ensuring the whole paraffin block fits into the field of view (reducing the size of the paraffin block or choosing a lower magnification). The artefact feature observed in the upper left part of Fig. 4(b) and upper right part of Fig. 4(f) is caused by phase wrapping at the edges of two air bubbles in the paraffin wax (clearly visible in the projections in Fig. 3).

**Fig. 5:**
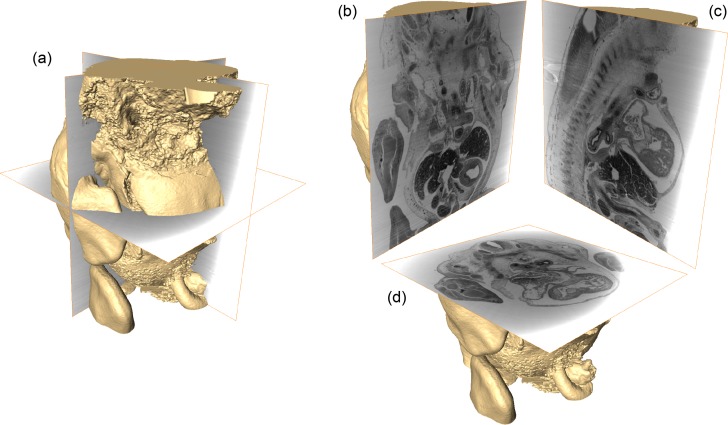
(a) 3D rendering of the mouse embryo extracted from the phase volume (orthogonal slicing planes through the centre are indicated). (b) Frontal, (c) sagittal and (d) transverse cuts through the embryo showing the corresponding slices through whole phase volume (including paraffin).

From the phase volume, we can extract the 3D shape of the mouse embryo from the surrounding paraffin by volume rendering. Figure 5(a) shows the rendered embryo and the frontal, sagittal and transverse slicing planes through the centre of the sample (grey areas show paraffin). The limbs and outer shape of the specimen are nicely visualised. Furthermore, it can be seen that the removal of the head and embedding in paraffin led to slight damage of the embryo skin and cracking of outer areas, which is also visible in Figs. 4(d) and 4(f). In Figs. 5(b)–5(d) cuts of the embryo and the corresponding central slices of the phase volume are shown. Animations sliding through all frontal, sagittal and transverse slices of the embryo volume can be found as supplementary files (see Visualization 1, Visualization 2, and Visualization 3).

The volume of the propagation-based signal, reconstructed from the same data set as the phase volume, is included in Fig. 8 in the Appendix in Sec. 6.1 for completeness. It is mainly dominated by edge enhancement fringes occurring in this imaging regime, as already observed in the projections. Phase retrieval was not performed as the propagation-based signal is not the focus of this work. Note that this is not an absorption volume as it would be obtained from a scan optimised for the retrieval of the imaginary part *β* of the refractive index. It has, however, been demonstrated in the literature that the phase signal surpasses the absorption signal in image quality when comparing measurements performed with separate setups optimised for the two imaging modalities [24].

**Fig. 6:**
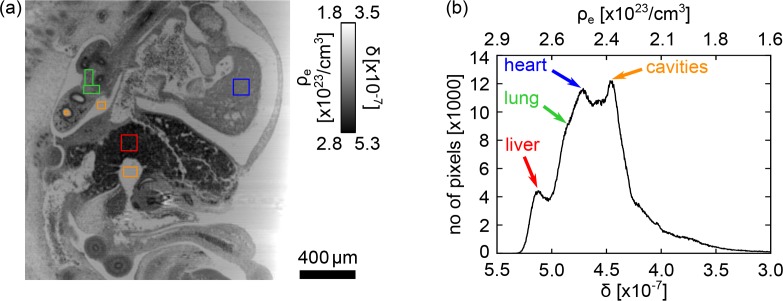
(a) Region of interest from the sagittal slice through the phase volume in Fig. 4 containing the organs of the mouse embryo. Small areas within the organs are chosen to determine the corresponding *δ*-values (coloured boxes). (b) Histogram of the slice in (a) showing a separation of the peaks for the different organs.

**Fig. 7:**
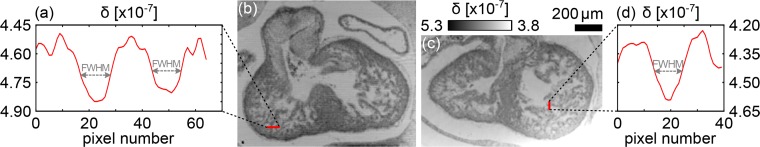
Region of the phase volume showing the ventricles of the mouse embryo heart in (b) transverse and (c) frontal view. Line profiles across the smallest discernible structures in (a) the transverse slice and (d) the frontal slice. The full width at half maximum of the peaks (FWHM ≈ 12 *μ*m) is taken as a measure of spatial resolution.

**Fig. 8:**
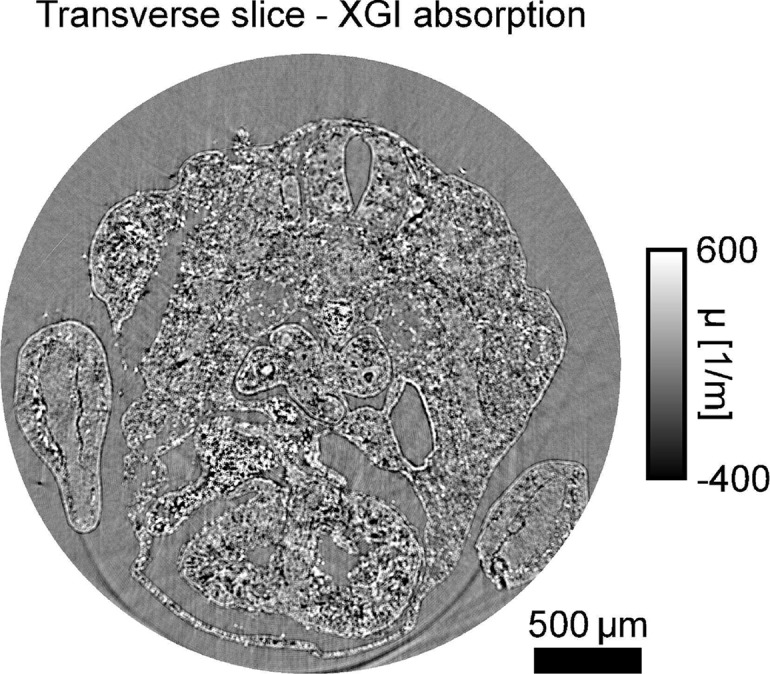
Transverse slice through the propagation volume showing a combination of absorption and edge-enhancement effects, retrieved from the same data set as the phase volume in Fig. 4. Negative values are due to edge enhancement leading to values larger than one.

## 4. Discussion

### 4.1. Qualitative and quantitative analysis of the XGI phase volume

As observed in the previous section, the phase signal shows the internal structure of the specimen in great detail due to its high sensitivity to small density differences. A qualitative assessment of the reconstructed phase volume can be conducted by visual inspection of the slices. The small electron density differences between the various types of tissue are clearly visualised in the phase signal in Figs. 4(b), 4(d) and 4(f) showing the organs of the mouse embryo with high contrast.

An advantage of XGI is that it provides quantitative information about the refraction and absorption properties of the sample under study. The quantity reconstructed in the phase tomogram is the distribution of the decrement *δ* of the complex refractive index or the electron density *ρ_e_*. To illustrate the separation of the small density differences in the sample, a histogram is plotted in Fig. 6(b), showing the distribution of *δ* and *ρ_e_* in a ROI of the sagittal slice of the phase volume containing mainly organs (see Fig. 6(a)). Regions of 10000 pixels centred in the different organs in Fig. 6(a) were chosen (coloured boxes) to determine an estimate for the *δ* values of the organs. Values were calculated for liver (red), lung (green), heart ventricles (blue) and the cavities inside the mouse embryo (orange) and are listed in Table 1. The organs of the embryo can be identified by the peaks of the histogram indicated with arrows in Fig. 6(b), which confirms the good contrast of the phase volume.

**Table 1:** Refractive index decrements *δ* and electron densities *ρ_e_* of the different organs of the mouse embryo measured in the small regions of interest (coloured boxes) in Fig. 6(a).

Organ	Liver	Lung	Heart	Cavities
*δ*[×10^−7^]	5.17 ± 0.03	4.84 ± 0.07	4.71 ± 0.04	4.44 ± 0.03
*ρ_e_*[×10^23^/cm^3^]	2.71 ± 0.02	2.53 ± 0.04	2.47 ± 0.02	2.32 ± 0.02

For further quantitative analysis the *δ*-values for liver and paraffin wax were determined from the whole phase volume by calculating the mean value in three regions of interest of 100×100×100 pixels located within the material. For liver a mean value of *δ*_liver_ = (5.10±0.05)× 10^−7^ is determined, which agrees within the error margins with the result retrieved from the 2D ROI in the sagittal slice listed in Table 1. For paraffin a value of *δ*_paraffin_ = (4.15 ± 0.18) × 10^−7^ is obtained in an area close to the sample. A theoretical value for *δ* of liver tissue can be derived from the properties of a liver phantom used in radiotherapy (Liver Equivalent Electron Density Plug of the Electron Density Phantom Model 062M supplied by Computerized Imaging Reference Systems, Inc., see www.cirsinc.com). The nominal electron density of the liver equivalent is *ρ*_*e* liver_ = 3.516 × 10^23^ electrons/m^3^, which can be converted to *δ*_liver, 19keV_ = 6.71 × 10^−7^ using Eq. (3). A theoretical value of *δ*_paraffin, 19keV_ = 6.26 × 10^−7^ for paraffin is obtained from [47] using the chemical formula C_25_H_52_ and density *ρ* = 0.95 g/cm^3^. The experimentally determined values are in the same order of magnitude as the theoretically calculated ones. However, a discrepancy from the literature values can be observed for both liver and paraffin to a similar degree. This can be partly caused by the changes in the properties of the sample induced by the effects of preparation and embedding. To separate the influence of the setup from the sample properties, a calibration sample could be used in future experiments. Moreover, phase-wrapping artefacts and the effects of region-of-interest tomography – as discussed in Sec. 3.3 – strongly affect the quantitative measurement of the refraction index decrement and lead to a deviation from the literature values. In future studies this will be prevented by scanning the sample in a water bath and adjusting the sample size and optical magnification to avoid region-of-interest tomography.

The spatial resolution of the reconstructed projection images is affected by the presence of the optical elements in the X-ray path, the resolution of the detector system, and the separation of the diffracted beams – created by the phase grating – in the observation plane. In this case the separation was 2 *λz/p* ≈ 9.4 *μ*m with the wavelength *λ* = 0.065 nm, the grating-detector distance *z* = 0.72 m, and the grating period *p* = 10 *μ*m. To estimate the upper limit of the spatial resolution in the reconstructed phase volume after the FBP step, we use the full width at half maximum (FWHM) of the smallest discernible structures in the phase volume slices, as proposed in [48]. Structures in transverse and frontal slices of the heart were chosen for this analysis and the selected features are indicated by red lines in Figs. 7(b) and 7(c), respectively. From the corresponding line profiles in Figs. 7(a) and 7(d) a FWHM of 11.8 *μ*m was measured for the transverse slice and FWHM values of 12.7 *μ*m and 12.3 *μ*m for the first and second feature (first and second peak) in the frontal slice.

### 4.2. Comparison of XGI and HREM results

As illustrated in Fig. 4, the quality of the phase tomogram retrieved in this study is comparable to the results obtained with the HREM method commonly used for this type of sample. While HREM can achieve excellent results, XGI has the major advantages of being a non-destructive technique and not requiring any contrast agents while delivering a similar image quality at a comparable scan time. As the specimen is not treated with staining solutions and is not physically sectioned, tissue distortions are minimised and the sample preparation time is significantly reduced. In contrast to HREM, XGI delivers quantitative volumetric information about the sample properties important e.g. for studies of diseases at different stages and developmental defects, for which quantitative density and volume measurements of the organs and tissue types are of interest.

## 5. Conclusions

We have demonstrated the high potential of X-ray grating interferometry with a single grating in phase-stepping mode for X-ray phase-contrast imaging of biological samples and have evaluated the results against data obtained from high-resolution episcopic microscopy measurements.

The single-grating XGI setup installed at I13-2 Beamline at Diamond Light Source showed high performance and stability. The phase volume of a mouse embryo embedded in paraffin wax obtained with this instrument reveals fine details of the organs at high contrast and resolution, comparable with state-of-the-art HREM images. Data acquisition can be performed in the short period of a few hours, maximising sample throughput and statistics of the results. As XGI with a single grating is a non-destructive method without the need for contrast agents, which can be implemented with a simple, robust and flexible setup, it provides a promising alternative to HREM for the 3D visualisation of the inner structure of biological specimens, especially for quantitative density and volume investigations.

For future studies the current setup will be optimised further to increase the visibility of the interference pattern, avoid artefacts induced by region-of-interest tomography, phase wrapping and optics instabilities and to quantify the effect of sample preparation. Moreover, it will be possible to speed up the data collection process by using a multilayer monochromator or polychromatic beam allowing shorter exposure times through higher photon flux and by optimising the data acquisition scheme, e.g. by taking the rotation axis as fast tomographic axis or employing an interlaced stepping method [49].

The high-quality images obtained with X-ray single-grating phase-contrast imaging will allow conducting high-throughput morphological, pathological and diagnostic investigations of biomedical specimens as an alternative or complementary method to HREM.

## 6. Appendix

### 6.1. Propagation volume

From the propagation projections, a volume visualising absorption and edge-enhancement effects in the sample was reconstructed. Figure 8 shows a transverse slice at the same position in the sample as the for the phase volume in Fig. 4(d).

### 6.2. Supplementary videos

Animations sliding through the frontal, sagittal and transverse slices of the phase volume can be found as additional files, see Visualization 1, Visualization 2, and Visualization 3.
